# Explanation

**Published:** 1895-04

**Authors:** 


### EXPLANATION.

The serious illness of the senior editor through the entire month of February prevented him from taking his usual place in the editorial pages. Our readers lost nothing by this, and he wishes here to express his grateful thanks to his colleagues for their timely aid under trying circumstances.

